# Multidisciplinary strategies to treat severe hypoglycemia in hospitalized patients with diabetes mellitus reduce inpatient mortality rate: Experience from an academic community hospital

**DOI:** 10.1371/journal.pone.0220956

**Published:** 2019-08-08

**Authors:** Deepak Kana Kadayakkara, Priyadarshini Balasubramanian, Katherine Araque, Karri Davis, Fahad Javed, Pontea Niaki, Sachin Majumdar, Gregory Buller

**Affiliations:** 1 Department of Medicine, Bridgeport Hospital-Yale New Haven Health, Connecticut, United States of America; 2 Department of Medicine, Yale School of Medicine, New Haven, Connecticut, United States of America; 3 Adult Endocrinology Department, National Institute of Diabetes and Digestive and Kidney Diseases, National Institutes of Health, Bethesda, Maryland, United States of America; Azienda Ospedaliero Universitaria Careggi, ITALY

## Abstract

**Objective:**

Severe hypoglycemia (blood glucose < 50 mg/dl) in hospitalized patients with diabetes mellitus is associated with poor outcomes such as increased mortality and readmission rates. We study the effects of system based interventions in managing severe hypoglycemia and its impact on outcomes.

**Research design and methods:**

We performed retrospective review of pre- and post- intervention study to quantify severe hypoglycemia in patients admitted in the general internal medicine wards with primary or secondary diagnosis of diabetes mellitus based on ICD-9 and ICD-10 codes. We implemented multidisciplinary interventions including standardization of treatment, education of in-patient medical teams and physician notification and feedback immediately after severe hypoglycemia. The endpoints were the comparative analysis of incidence of severe hypoglycemia, in-patient mortality rate, 30-day mortality rate, 30-day readmission rate, recovery time from hypoglycemia, time to next glucose measurements, use of standardized treatment and physician notification rate pre-and post-intervention.

**Results:**

The incidence of severe hypoglycemia per patient with diabetes was reduced from 9.6% (233/2416) to 5.6% (202/3607) (p<0.001) post-intervention. The in-patient mortality rate in patients with severe hypoglycemia reduced from 4.1% to 0% (p = 0.019), 30-day mortality rate reduced from 9.8% to 3.8% (p = 0.058) post-intervention. 30-day readmission rate was comparable between pre-intervention (31.7%) and post-intervention (29%) (p = 0.60). In comparison, the mortality and readmission rates of all diabetic patients did not reduce during the same observation periods. Recovery time reduced from 116 (83–161) to 75 (57–102) min (p<0.01), time to next glucose measurement reduced from 39.5 (34–48) to 32 (28–35) min (p<0.01), use of standardized treatment improved from 22.7% (53/233) to 72.2% (146/202) (p<0.001) and physician notification rate increased from 29.2 (68/233) to 84.7% (171/202) post-intervention.

**Conclusions:**

Our study shows that multidisciplinary strategies improves the process of early detection and management of severe hypoglycemia and reduce incidence and in-patient mortality rate.

## Introduction

Severe hypoglycemic episodes (blood glucose < 50 mg/dl) in hospitalized patients is associated with increased inpatient mortality [1–3], readmission rate [4, 5] and mortality following one year after discharge [5]. Serious adverse effects of severe hypoglycemia are ventricular tachycardia and seizures that could lead to long term neurological sequelae with cognitive decline or death [6, 7]. The incidence of severe hypoglycemia in hospitalized patients ranges from 7 to 35% [5, 8–10]. In hospitalized patients who were treated with insulin, inpatient mortality occurred in 7.6% of hospitalizations with severe hypoglycemia and 3.8% in those without [1]. The analysis of link between severe hypoglycemia and mortality in Action to Control Cardiovascular Risk in Diabetes (ACCORD) trial participants has shown that annual mortality rates were higher in patients who had one or more episodes of hypoglycemia compared to patients who did not experience hypoglycemia in both intensive glucose control arm (mortality rate of 2.8% in hypoglycemia and 1.2% without hypoglycemia) and standard glucose control arm (mortality rate of 3.7% in hypoglycemia and 1% without hypoglycemia) [3].

The literature is abundant with studies recognizing the short-term and long term sequelae of hypoglycemia. However, evidence based interventions that prevent hypoglycemia and the effectiveness of multidisciplinary hypoglycemia treatment from a hospital system perspective are scarce [11–13]. These studies primarily focus on improving the process of hypoglycemia detection and treatment, as the first step to understand the impacts of severe hypoglycemia in patients with diabetes. We have previously shown that by implementing our proposed multidisciplinary strategies, the time to recovery from severe hypoglycemia to euglycemia (>100 mg/dl) and the time to next finger stick glucose measurements after severe hypoglycemia can be decreased, and physician notification rate and standardized treatment can be improved [13]. The impact of the system-based interventions of severe hypoglycemia management on outcomes such as mortality and readmission has not been studied before.

It is unclear if hypoglycemia is a biomarker of morbidity and mortality that reflect the severity of the underlying illnesses or if it directly contributes to morbidity and mortality. Here, we describe the impact of system-based hypoglycemia management on outcomes in an academic community hospital.

## Materials and methods

### Data collection

We performed retrospective review of pre- and post- intervention study of severe hypoglycemia in all hospitalized patients in Bridgeport Hospital, an affiliated academic hospital in the Yale New Haven health system with 383 beds. The study was conducted according to IRB approval and it involved retrospective review of charts. Informed consent was waived by the IRB committee. Patients with primary or secondary diagnosis of diabetes mellitus (based on ICD-9 and ICD-10 codes) admitted in the general medical wards were included in the study. Patients admitted or transferred to the intensive care units, psychiatry wards, and OB/GYN wards were excluded. The patients in the ICU were excluded because ICU had a separate protocol for hypoglycemia management. The data were collected from EPIC electronic medical records. The time frame of the study was September 2013 to September 2016 of which September 2013 to May 2014 was the pre-intervention period and January 2016 to September 2016 was the post-intervention period. The intervention was implemented in all the medical wards in phases starting from June 2014 and completed in December 2015.We collected demographic information such as age, gender, body mass index and renal function. We recorded the date and time of severe hypoglycemia, the treatment response, time of follow up finger stick glucose measurements, modes of treatment, time of euglycemia (BG>100 mg/dl) and change in treatment strategy following hypoglycemia. Serum glucose obtained from basic metabolic panel and point of care finger stick glucose measurements were included in the study. We calculated the recovery time from severe hypoglycemia to euglycemia, time to next follow up finger stick glucose measurements, in-patient mortality, 30-day mortality and 30-day readmission rates.

### Designing of interventions

Design of interventions has been described in detail in our first report [13]. In summary, an interdisciplinary committee including physicians, nurses, educators, pharmacists and clinical redesign specialist were formed and a set of intervention known as hypoglycemia bundle of care was designed. The hypoglycemia bundle of care was designed based on root cause analysis of the entire process of hypoglycemia detection, reporting and management. The components of root cause analysis were factors influencing the process, equipment and supplies, communication and staffing. System based interventions were designed based on the root cause analysis and it included standardization of treatment using either glucose gel, intravenous dextrose or intramuscular glucagon, dextrose administration of nursing staff without requirement of physician orders and Pyxis Medstation alert to check follow up glucose after treatment, EMR order set for insulin regimen along with automated hypoglycemia order set, automated physician notification immediately after hypoglycemia to reassess risks, automated best practice advisor on EMR with recommendations for endocrine consultation after two severe hypoglycemic events, standardized hypoglycemia management protocol available as a laminated card attached to staff ID badge and education of inpatient medical teams. If an incidence of severe hypoglycemia is reported the staff was instructed to check glucose values every 15 minutes until euglycemia is achieved (100 mg/dl). Glucose value of 100 mg/dl was chosen as the target for euglycemia arbitrarily because glucose values of 100 mg/dl and above would be safe glucose levels where patients can be left on the ward without checking glucose values every 15 minutes.

### Outcome measurements

Outcomes measured included incidence of severe hypoglycemia, in-patient mortality, 30-day mortality and 30-day readmission rates. The efficiency of the process improvement was measured by calculating recovery time from severe hypoglycemia to euglycemia and time to next follow up finger stick glucose measurements, physician notification rate, change in treatment regimen and use of standardized treatment like IV dextrose, glucose gel and glucagon.

### Statistical analysis

Histogram analysis of our data showed that the data were not normally distributed. We used non-parametric Mann-Whitney U test for continuous variables and Chi-Square test for categorical variables and proportions.

## Results

There were 2416 patients admitted with diabetes during the 9 months pre-intervention period and 3607 patients in the 9 months post-intervention period. We identified 233 severe hypoglycemia in 123 patients pre-intervention and 202 severe hypoglycemia in 131 patients post intervention. The demographic data are represented in Table 1.

**Table 1:** Demographic data.

	Pre-intervention	Post-intervention	P value	Statistical test
No. of all diabetic patients	2416	3607		
Female/Male	1158/1258	1722/1885	0.88	Chi-Square
Age (Median, 95% CI) yrs	67 (66–67)	67 (67–68)	0.16	Mann-Whitney
No. of patients with severe hypoglycemia	123	131		
Age (Median, 95% CI) yrs	67 (63–70)	66 (64–70)	0.77	Mann-Whitney
Female/Male	76/47	70/61	<0.001	Chi-Square
BMI (Median, 95% CI) kg/m^2^	26.58 (25.16–27.3)	26.69 (25.68–28.06)	0.69	Mann-Whitney
Creatinine (Median, 95%CI) mg/dl	1.44 (1.22–1.56)	1.5 (1.26–2.29)	0.16	Mann-Whitney

The incidence of severe hypoglycemia per patient admitted with diabetes in the pre-intervention period was 9.6% (233/2416), which reduced to 5.6% (202/3607) post-intervention (p<0.001). The in-patient mortality of all patients with diabetes in the pre-intervention period was 1.86% (45/2416) and 2.25% (81/3607) in the post-intervention period (p = 0.14). However, the in-patient mortality rate of patients with severe hypoglycemia reduced significantly in the post-intervention period (p = 0.019). In the pre-intervention period, the in-patient mortality was 4.07% (5/123) and in the post-intervention phase it was 0% (0/131). In addition to in-patient mortality, the 30 day mortality and 30 day readmission rate are tracked as hospital quality and safety measures. The 30-day mortality rate of all diabetic patients remained unchanged during pre- and post-intervention (p = 0.76). The 30-day mortality rate of all diabetic patients were 5.92% (143/2416) pre-intervention and 5.74% (207/3607) in the post-intervention period. However, in patients with severe hypoglycemia, 30-day mortality reduced from 9.72% (12/123) to 3.82% (5/131) (p = 0.058). The in-patient mortality and 30-day mortality rates of patients with severe hypoglycemia were higher in the pre-intervention period compared to mortality rates in all diabetic patients. This observation is consistent with studies published before [1, 3]. During the post-intervention period, however, the mortality rate of severe hypoglycemia patients is comparable to all diabetic patients reflecting the impact of our interventions. As observed with the mortality rates, 30-day readmission rate in all diabetic patients did not change during the pre-intervention period, 25.17% (608/2416) and post intervention period, 24.09% (869/3607) (p = 0.73). In patients with severe hypoglycemia, there was decrease in 30-day readmission rates post-intervention, however was not statistically significant (p = 0.6). The 30-day readmission rate of patients with severe hypoglycemia in the pre-intervention period was 31.71% (39/123) and it 29% (38/131) in the post-intervention period. The results are summarized in Table 2.

**Table 2:** Outcome measures.

	Pre-intervention	Post-intervention	P value	Statistical test
Incidence rate of severe hypoglycemia per diabetic admission	233/2416 (9.6%)	202/3607 (5.6%)	<0.001	Chi-square
In-patient mortality in all diabetes patients	45/2416 (1.86%)	81/3607 (2.25%)	0.14	Chi-square
In-patient mortality in severe hypoglycemia	5/123 (4.07%)	0/131 (0%)	0.019	Chi-square
30-day mortality all diabetes patients	143/2416 (5.9%)	207/3607 (5.7%)	0.76	Chi-square
30-day mortality in severe hypoglycemia	12/123 (9.72%)	5/131 (3.82%)	0.058	Chi-square
30-day readmission all diabetes patients	608/2416 (25.17%)	869/3607 (0.73%)	0.73	Chi-square
30-day readmission severe hypoglycemia	39/123 (31.71%)	38/131 (29%)	0.6	Chi-square

In our published pilot study in two hospital wards, we showed that after the interventions, significant improvement can be achieved in process related outcomes such as time to recovery from severe hypoglycemia to euglycemia (BG>100 mg/dl), time to next follow up finger stick glucose measurements, use of standardized hypoglycemia treatment and physician notification [13]. In this study, we have expanded the implementation of the hypoglycemia bundle to all medical wards in the hospital. The recovery time to euglycemia and time to next finger stick glucose indicate the effectiveness of the process improvement achieved by our interventions. The median time to recovery was significantly reduced from 116 (95% CI: 83–161) min in the pre-intervention period to 75 (95% CI: 57–102) min in the post-intervention period (p<0.0076). And the median time to next finger stick glucose was significantly reduced from 39.5 (95% CI: 34–48) min to 32 (95% CI: 28–35) min (p = 0.0017) post-intervention. Physician notification rate improved from 29.2% (68/233) to 84.7% (171/202) (p<0.0001), and the rate of standardized hypoglycemia treatment improved from 22.7% (53/233) to 72.2% (146/202) (p<0.0001) post-intervention respectively. Based on the data, physicians are taking responsibility for making changes to the treatment regimen to prevent further episodes of severe hypoglycemia. The diabetes treatment regimen was modified in 40.7% of the severe hypoglycemia episodes during the pre-intervention period and in 60.8% of the severe hypoglycemia episodes in the post-intervention period (p<0.0001). The results are summarized in Table 3.

**Table 3:** Process improvement measures.

	Pre-intervention	Post-intervention	P value	Statistical tests
Recovery time (min) (median, 95% CI)	116 (83–161)	75 (57–102)	0.0076	Mann-Whitney
Time to next FS (min) (median, 95% CI)	39.5 (34–48)	32 (28–35)	0.0017	Mann-Whitney
Physician notification rate	68/233 (29.2%)	171/202 (84.7%)	<0.0001	Chi-Square
Standardized treatment (IV dextrose or glucose gel)	53/233 (22.7%)	146/202 (72.2%)	<0.0001	Chi-Square
Modification in anti-diabetes regimen following severe hypoglycemia	95/233 (40.7%)	123/202 (60.8%)	<0.0001	Chi-square

## Discussion

This study analyzes how a multidisciplinary system-based intervention for hypoglycemia management in hospitalized patients with diabetes mellitus could improve outcomes. Although hypoglycemia is recognized as a complication of hospitalization that results in increased mortality and readmission rates [1–5], the impact of treating hypoglycemia on the outcomes has not been studied before. In-patient mortality, 30-day mortality and 30-day readmission rates are common benchmarks used by hospitals in determining the quality and safety of care. Based on our observations, our proposed hypoglycemia bundle of care reduces inpatient mortality rate and 30-day mortality rates suggesting that hypoglycemia management may have direct impact on outcomes. During the same period, the inpatient mortality rate and 30-day mortality rates in all diabetic patients did not change. The analysis of mortality in patients with hypoglycemia in reference to all hospitalized diabetic patients would eliminate potential biases that could have risen from the differences in the standard of hospital care at different points of time. We also show that the patients with severe hypoglycemia have higher mortality and readmission rates compared to all diabetic patients, which has been observed in prior reports [1–4]. The differences in the mortality rate between severe hypoglycemia patients and all diabetic patients narrows after the interventions suggesting that interventions directly impact mortality. Further studies may be required to elucidate the mechanistic aspects of these results.

It is important to note that the previous studies related to hypoglycemia management in hospitalized patients focused on improving the process of detection and management of hypoglycemia and not outcomes. While this reported literature has taught us how to improve the process of detection and multidisciplinary response to hypoglycemia, here we analyze the impact of these interventions on mortality outcomes. Multidisciplinary interventions included educating nurses and physicians, using standardized treatment for hypoglycemia such as IV insulin, glucose gel and glucagon, integrating insulin order sets and hypoglycemia treatment algorithm in electronic medical records, dextrose administration by nursing staff without physician orders and automated physician notification immediately after severe hypoglycemia to reassess risks. Following the interventions, we noticed significant improvement in all process-related measures such as time to recovery, time to next finger stick glucose measurements, physician notification rate and use of standardized treatment. These results are also consistent with previous approaches of system-based hypoglycemia management in hospitalized patients [11–14].

Our study is not without limitations. It is a relatively small single center observational study and may not be powered enough to identify the differences in all outcomes. Patients who were admitted in the ICUs were excluded from the study and that would result in the exclusion of critically ill patients. Despite these limitations, we believe that it could serve as a launching point for larger scale multi-center trials. Our proposed approach can in future, be studied in other hospitals to facilitate the recognition, management and aid healthcare providers to manage inpatient hypoglycemic events, improve patient safety and reduce mortality rates in patients with diabetes that require inpatient care.

## Conclusion

Severe hypoglycemia in hospitalized patients with diabetes increased morbidity and mortality. Previous studies on inpatient hypoglycemia management has focused primarily on the process of early detection and management. In this study, we show that multidisciplinary system-based interventions for severe hypoglycemia management not only improve the processes, but can improve health care outcomes, incidence of severe hypoglycemia, inpatient mortality and 30-day mortality rates.
