# Correction: Multidisciplinary strategies to treat severe hypoglycemia in hospitalized patients with diabetes mellitus reduce inpatient mortality rate: Experience from an academic community hospital

**DOI:** 10.1371/journal.pone.0222475

**Published:** 2019-09-06

**Authors:** Deepak Kana Kadayakkara, Priyadarshini Balasubramanian, Katherine A. Araque, Karri Davis, Fahad Javed, Pontea Niaki, Sachin Majumdar, Gregory Buller

The third author's name is spelled incorrectly. The correct name is: Katherine A. Araque. The correct citation is: Kana Kadayakkara D, Balasubramanian P, Araque KA, Davis K, Javed F, Niaki P, et al. (2019) Multidisciplinary strategies to treat severe hypoglycemia in hospitalized patients with diabetes mellitus reduce inpatient mortality rate: Experience from an academic community hospital. PLoS ONE 14(8): e0220956. https://doi.org/10.1371/journal.pone.0220956
